# Enhanced bacterial clearance in early secondary sepsis in a porcine intensive care model

**DOI:** 10.1038/s41598-023-28880-x

**Published:** 2023-02-03

**Authors:** Frida Wilske, Paul Skorup, Katja Hanslin, Helena Janols, Anders Larsson, Miklós Lipcsey, Jan Sjölin

**Affiliations:** 1 Section of Infectious Diseases, Department of Medical Sciences, Uppsala University, SE 751 85 Uppsala, Sweden; 2 Section of Anaesthesiology and Intensive Care, Department of Surgical Sciences, Uppsala University, Uppsala, Sweden; 3 Section of Clinical Chemistry, Department of Medical Sciences, Uppsala University, Uppsala, Sweden; 4 Hedenstierna Laboratory, Anaesthesiology and Intensive Care, Department of Surgical Sciences, Uppsala University, Uppsala, Sweden

**Keywords:** Immunology, Microbiology, Infectious diseases, Bacterial infection

## Abstract

Early secondary sepsis (ESS), occurring after recent inflammatory activation is associated with a reduced inflammatory response. If this attenuation also is associated with decreased bacterial killing, the need for antibiotic efficacy might be greater than in primary sepsis (PS). This prospective, randomised interventional study compares bacterial killing in ESS and PS in a large animal intensive care sepsis model. 38 pigs were intravenously administered live *Escherichia coli *for 3 h. Before baseline ESS was pre-exposed to endotoxin 24 h, whereas PS was not. Bacterial growth was measured in organs immediately post-mortem, repeatedly during 6 h in blood in vivo and for blood intrinsic bactericidal capacity ex vivo. Splenic growth was lower in ESS animals, than in PS animals (3.31 ± 0.12, vs. 3.84 ± 0.14 log_10_ CFU/mL, mean ± SEM) (*p* < 0.01) with a similar trend in hepatic growth (*p* = NS). Blood bacterial count at 2 h correlated with splenic bacterial count in ESS (ESS: r = 0.71, *p* < 0.001) and to blood killing capacity in PS (PS: r = 0.69, *p* < 0.001). Attenuated inflammation in ESS is associated with enhanced antibacterial capacities in the spleen. In ESS blood bacterial count is related to splenic killing and in PS to blood bactericidal capacity. The results suggest no increased need for synergistic antibiotic combinations in ESS.

## Introduction

Sepsis is an infection that leads to life-threatening organ dysfunctions caused by a dysregulated host response^1^. Current studies report simultaneously increased production of pro- and anti-inflammatory cytokines^2^. Most of sepsis-related deaths are not caused by a pro-inflammatory cytokine storm but occur later during a prolonged immunosuppressive phase, probably because of failure to control the primary infection or subsequent hospital-acquired infections^3,4^. Early secondary sepsis (ESS), here defined as a sepsis that develops in a situation of recent inflammatory activation with features of the well-studied endotoxin tolerance, is in experimental animal and human studies associated with attenuated inflammatory responses^5–8^. This mitigated response is further linked to less organ dysfunction which has been observed in an experimental porcine model and in a retrospective study in patients in an intensive care unit (ICU)^5,9^. Adding an aminoglycoside or quinolone to a beta-lactam antibiotic to achieve a synergistic effect in the initial sepsis treatment is controversial. Clinical studies have resulted in varying outcomes^10,11^ and even high-quality reviews such as the Surviving Sepsis Campaign and Infectious Diseases Society of America (IDSA) initially reached different conclusions depending on how the studies were assessed, although their opinions now are more concordant^12–14^. One possible explanation for the divergent results could be that patients with sepsis have different demands of antibiotic efficacy and synergy. If the reduced inflammatory response following recent activation is also associated with decreased bacterial killing, it may be that the effect of a synergistic antibiotic regimen will be greater in these patients than in those with primary sepsis (PS) without such prior activation.

To answer this question the first issue will be to study the bacterial clearance in a model of endotoxin tolerance. Only a few studies exist in which sepsis caused by live bacteria represent the second challenge and there are no data from humans or large animals. An improved bacterial clearance was demonstrated in mice^6,15,16^. However, there are difficulties extrapolating these results to humans because of a similarity to the human immune system of only 20%^17,18^. Furthermore, contradictory results have been reported in an endotoxin-tolerant rabbit model^19^. A study in humans would be challenging and unethical, which is why we explore this question in a porcine endotoxin tolerant large animal model that has similarities to human sepsis^17,18^ and includes intensive care measures known to additionally affect the inflammatory response^5^.

Thus, the primary aim of the present study was to compare the bacterial killing in pigs with ESS after exposure to a 24-h endotoxin challenge and intensive care treatment with that in unexposed animals with PS. Secondary aims were to analyse the relationships between blood bacterial count during infusion and blood intrinsic bactericidal capacity before and after the bacterial challenge and bacterial growth in organs.

## Material and methods

### Anaesthesia, preparations and intensive care settings

The animals, 38 healthy Norwegian landrace-breed piglets of both sexes, were used. The preferred weight was 25 kg giving an age of 9–12 weeks, thus, the animals were not sexually mature. They were prepared, anaesthetised and handled as previously reported^20^ and described in Supplemental Digital Content 1. Briefly, mechanical ventilation and intravenous (iv) general anaesthesia were employed and the intensive care was maintained using an intensive care treatment protocol designed to keep mean arterial pressure, cardiac output, arterial partial pressure of oxygen and carbon dioxide and blood glucose within specified limits (Supplemental Digital Content 2).

### Protocol/experimental design

**Figure 1:**
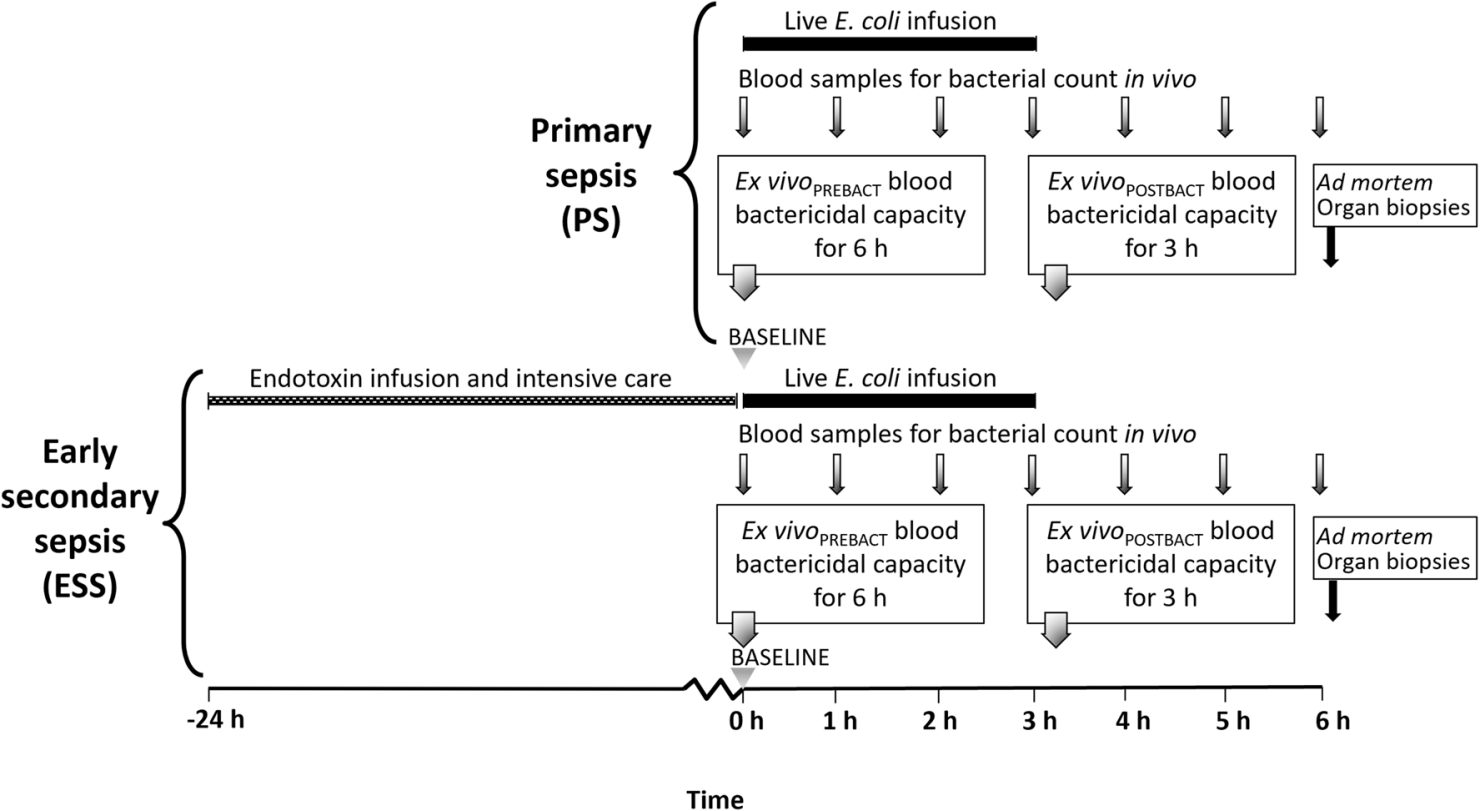
Schematic presentation of the study and the primary and early secondary sepsis groups. ESS animals (n = 21) were pre-exposed to 24 h endotoxin infusion and intensive care treatment before baseline, whereas PS animals (n = 17) were unexposed at baseline when the experiment was initiated by a 3-h intravenous infusion of live *E. coli*. Bars represent infusions while arrows denote time points for sampling for bacterial analyses.

The experimental design is illustrated in Fig. 1. The animals were randomised to either the ESS group in which animals were exposed to an endotoxin infusion and intensive care for 24 h before baseline or the PS group, in which unexposed animals were challenged with live bacteria at baseline. At baseline, the 6 h experiment was initiated with a 3-h iv infusion of *E. coli*, followed by a 3-h observation period before sacrificing the animals by a potassium chloride injection. Immediately before baseline and repeatedly during the experiment, physiological data were recorded. Blood samples for analysis of cytokines and Sequential Organ Failure Assessment (SOFA) were taken hourly. Organ samples were taken within 30 min *post-mortem*.

### Endotoxin

The endotoxin in the ESS group consisted of a lipopolysaccharide from *E. coli,* 0111:B4; (Sigma Chemical Co., St Louis MO, USA) and the same batch was used to all animals to minimise batch variations. The endotoxin infusion was given to establish endotoxin tolerance which has previously been reported in this porcine model^5^: after a starting dose of 0.3 μg × h^−1^, a stepwise increase for 30 min ×followed until a final dose of 0.063 μg × kg^−1^ × h^−1^ was achieved. This dose was then continued until completion at 24 h after the start of the infusion.

### Organism

The *E. coli* strain B09-11822 (serotype O-rough:K1:H7; Statens Seruminstitut, Copenhagen, Denmark) is an encapsulated and serum-resistant clinical isolate. The preparation of the bacteria is described in Supplemental Digital Content 3.

### Measurements

#### In vivo blood and organ bacterial cultures

Bacterial investigations from arterial blood, spleen and liver are described in Supplemental Digital Content 4. Other organs than the spleen and liver, primarily the kidney and lung were also investigated in most animals, but with too few bacteria found to allow for a meaningful analysis.

#### Blood intrinsic bactericidal capacity

The blood intrinsic bactericidal capacity was analysed by ex vivo bacterial clearance. Arterial blood was obtained at baseline before bacterial infusion (ex vivo_PREBACT_) and 15 min after termination (ex vivo_POSTBACT_) and ex vivo inoculated with *E. coli* at a concentration of 10^5^ CFU × mL^−1^. The ex vivo_PREBACT_ incubation lasted for 6 h and ex vivo_POSTBACT_ for 3 h with bacterial quantifications at 3 and 6 h. Bacterial clearance was calculated by subtracting the log bacterial count from that of the starting inoculum at 0 h. Due to a lack of time, ex vivo_PREBACT_ clearance was not achieved in two ESS animals and ex vivo_POSTBACT_ clearance in two PS animals.

#### Organ parameters and laboratory tests to evaluate the sepsis reaction, maintenance of intensive care and the inflammatory response

Details of blood test analysis, organ dysfunction parameters and cytokines are presented in Supplemental Digital Content 5. The sepsis reaction in the two groups was evaluated with the SOFA score^1^, although no assessment of the central nervous system was possible due to sedation. The inflammatory response was estimated by the highest levels of tumour necrosis factor-α (TNF-α) and interleukin-6 (IL-6) in plasma analysed by commercial porcine-specific sandwich enzyme-linked immunosorbent assays.

### Calculations and statistics

Because the bacterial clearance from the blood has been shown to be rapid, the primary endpoint of the study was bacterial growth in the spleen and liver, the two principal organs responsible for the elimination of bacteria^20^. With a standard deviation in the PS group of 20%, a power of 0.8, a two-sided *α*-error of 0.05 and a detectable difference of 20%, 17 animals were required. In the ESS group the standard deviation was expected to be 25% higher and with the same power, *α*-error and detectable difference 21 animals had to be included. In the analysis of the primary endpoint Student’s unpaired t-test was applied. Because ex vivo growth in blood did not follow a normal distribution, other differences between the groups were analysed by the Mann–Whitney U test, paired analysis by the Wilcoxon matched-paired test and correlations by the Spearman rank test. Normally distributed data are expressed as mean ± SD and non-normally as median and interquartile range (IQR). Statistica software (v13.5, StatSoft, Tulsa, OK, USA) was used to generate the calculations and *p* < 0.05 was considered statistically significant.

### Animals and ethic statements

This study was conducted after approval from the Animal Ethical Board in Uppsala, Sweden (permit no C250/11 and C155/14). Animals were handled in accordance with the Guide for the Care and Use of Laboratory Animals and reported in compliance with the ARRIVE guidelines^21^.

## Results

### Animal experiment

The animals developed sepsis or septic shock during the bacterial infusion with SOFA scores of 6 (3.5–8) in the ESS group and 9 (6.5–11) in the PS group. The dose of infused bacteria was 8.80 ± 0.10 in the ESS group and 8.80 ± 0.09 log_10_ CFU in the PS group. Body weights in the ESS and PS groups were 25.3 ± 1.9 and 25.2 ± 1.8 kg, respectively. Baseline at 0 h and the highest (peak) cytokine levels for TNF-α and IL-6 in the two groups are shown in Table 1 with significant reductions in the ESS animals (*p* < 0.001). The peak concentrations also appeared later in the ESS than in the PS group with TNF-α-peaking at 2 h compared with 1 h and IL-6 at 4 h compared with 3 h.

**Table 1:** Baseline at 0 h before the bacterial challenge and the highest (peak) plasma levels of TNF-α and IL-6 in animals with early secondary sepsis (ESS) and primary sepsis (PS). Data are presented as mean ± SD.

	ESS Log_10_ ng × L^−1^ (n = 21)	PS Log_10_ ng × L^−1^ (n = 17)
TNF-α		
Baseline	1.83 ± 0.27	1.87 ± 0.22
Peak	2.43 ± 0.45***	4.80 ± 0.31
IL-6		
Baseline	2.09 ± 0.35	1.85 ± 0.47
Peak	2.84 ± 0.36***	3.43 ± 0.31

Student’s unpaired t-test was used to compare the differences between ESS and PS.

***p < 0.001 ESS versus PS.

### Bacterial investigations

#### Organ bacterial cultures

**Figure 2:**
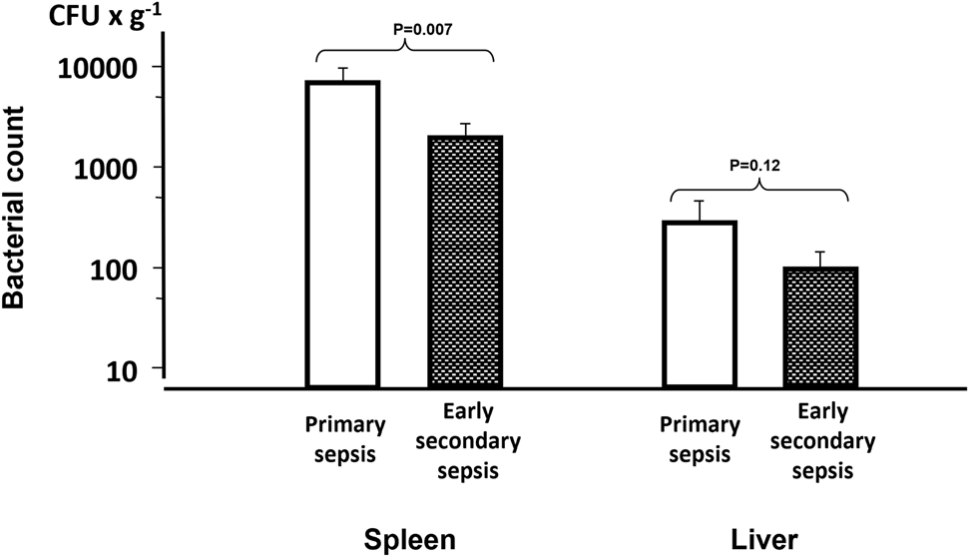
Bacterial count in the spleen and liver in primary (n = 17) and early secondary sepsis (n = 21). Values are mean ± SEM.

Organ bacterial cultures are depicted in Fig. 2. The ESS group exhibited lower growth in the spleen, than the PS group, (3.31 ± 0.56 vs 3.84 ± 0.56 log_10_ CFU × g^−1^) (*p* = 0.007). Bacterial growth in the liver was also lower in the ESS group than in the PS group (1.99 ± 0.78 vs 2.44 ± 1.00 log_10_ CFU × g^−1^), although the difference did not reach statistical significance.

#### Blood cultures in vivo

No animal exhibited *E. coli* growth in the blood culture before the bacterial infusion was initiated. The ESS animals showed a tendency to fewer bacteria in cultures during the bacterial infusions compared with the PS animals (Table 2). After termination of the infusion, no living bacteria were found in blood cultures.

**Table 2:** Bacterial growth in blood cultures obtained in vivo and blood intrinsic bactericidal capacity expressed as bacterial clearance ex vivo in blood obtained before the bacterial challenge at 0 h (Ex vivo_Prebact_) and 15 min after termination of the 3-h bacterial infusion (Ex vivo_Postbact_) in animals with early secondary sepsis (ESS) and primary sepsis (PS).

	Bacterial growth In vivo log_10_ CFU × mL^−1^		Bacterial clearance Ex vivo_PREBACT_ log_10_ CFU × mL^−1^		Bacterial clearance Ex vivo_POSTBACT_ log_10_ CFU ×mL^−1^	
	ESS (n = 21)	PS (n = 17)	ESS (n = 19)	PS (n = 17)	ESS (n = 21)	PS (n = 15)
Ex vivo growth 0 h			4.91 (4.86–4.97)	4.94 (4.67–4.96)	5.02 (4.99–5.12)	5.00 (4.86–5.06)
0 h	NG (NG–NG)	NG (NG–NG)	–	–	–	–
1 h	2.90 (2.48–3.02)	3.12 (2.92–3.24)				
2 h	3.14 (2.77–3.41)	3.21 (3.01–3.65)				
3 h	3.19 (2.88–3.55)	3.39 (3.22–3.75)	2.38 (1.52–2.99)	2.24 (1.22–3.22)	1.80 (0.91–3.16)^a^	0.36 (-0.64–2.52)
4 h	NG (NG–NG)	NG (NG–NG)				
5 h	NG (NG–NG)	NG (NG–NG)				
6 h	NG (NG–NG)	NG (NG–NG)	3.57 (2.00–4.20)	3.39 (1.35–3.98)		

Bacterial clearance is calculated as the difference between growth at 0 h and the different time points. Due to a lack of time, ex vivo_PREBACT_ analyses were not achieved in two ESS animals and in two PS animals in the ex vivo_POSTBACT_ analyses.

Values are median (interquartile range) log_10_ CFU × mL^−1^.

NG = No growth, detection limit 5 CFU × mL^−1^.

The Mann–Whitney U test was conducted to determine differences between the ESS and PS groups at each time point.

^a^*p* < 0.05 ESS versus PS.

#### Ex vivo bacterial clearance

Ex vivo results are summarised in Table 2. Blood intrinsic bactericidal capacity at baseline, expressed as ex vivo_PREBACT_ bacterial clearance at 6 h did not differ between the ESS and PS groups. A reduction in ex vivo_POSTBACT_ clearance compared with ex vivo_PREBACT_ clearance was found in the two groups (*p* = 0.004, n = 34). Moreover, the ex vivo_POSTBACT_ bacterial clearance in the PS group was lower than that in the ESS group (*p* = 0.028) with some animals not killing the bacteria.

#### Correlation analyses

**Figure 3:**
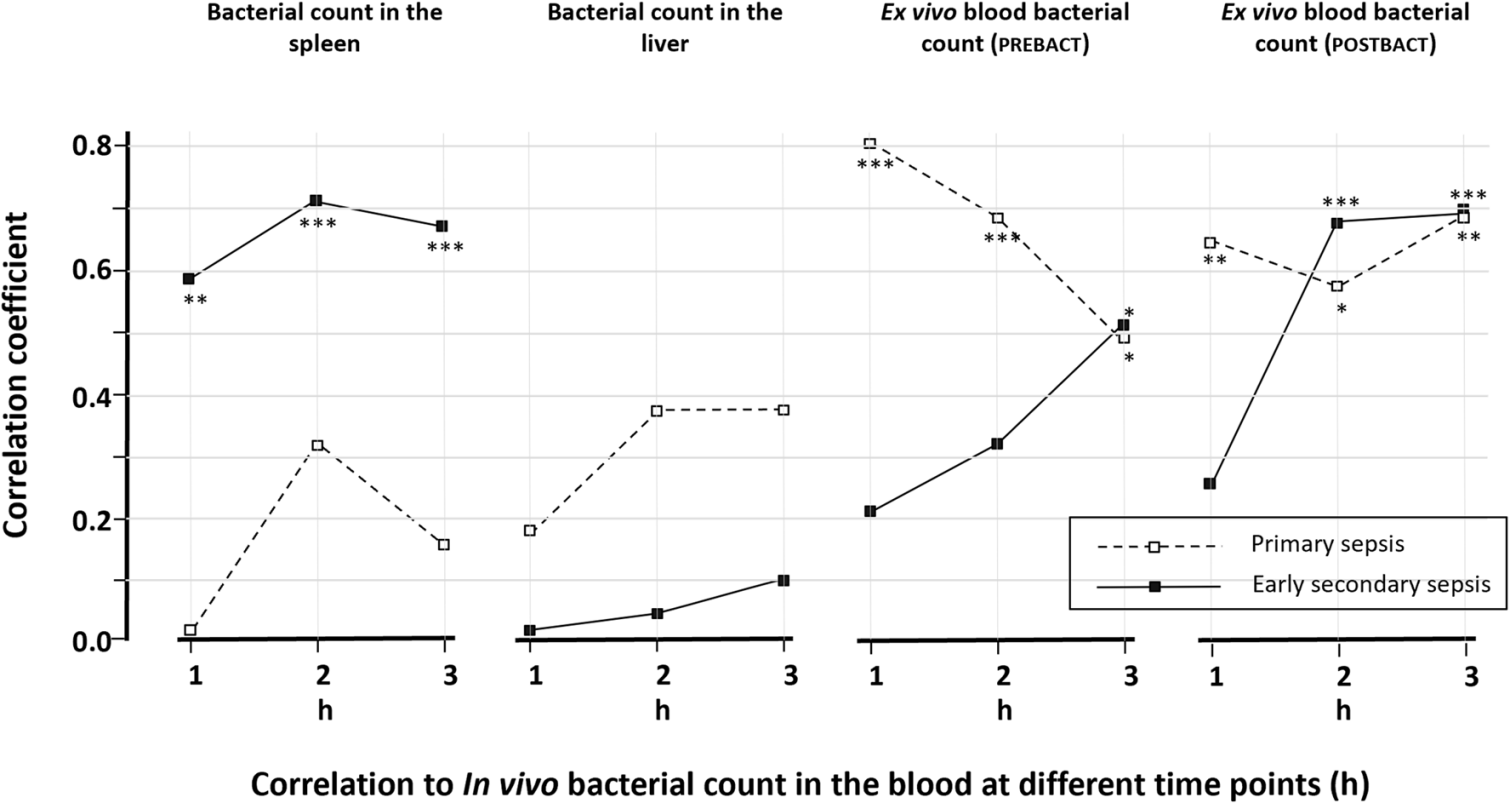
Spearman rank correlation analyses between in vivo bacterial count in the blood at 1–3 h and the bacterial counts in the spleen and liver, ex vivo_PREBACT_ bactericidal clearance at 6 h in blood before the bacterial challenge and ex vivo_POSTBACT_ bactericidal count at 3 h in blood after the bacterial challenge. The correlations between bacterial count in the blood and the two ex vivo clearances are negative but to relate to the positive values on the y-axis, these correlations are expressed as ex vivo bacterial count at the last determination after 6 and 3 h, respectively. *p < 0.05, **p < 0.01, ***p < 0.001.

A significant correlation was observed between bacterial growth in the spleen and liver in the PS group (r = 0.69, *p* = 0.003), but not in the ESS group (r = 0.26, *p* = NS). Correlations between bacterial in vivo count in the blood at 1–3 h and the bacterial count in the spleen and liver are depicted in Fig. 3. In the ESS group, there was a strong correlation between growth in the blood and spleen over all 3 h (r ranging 0.58–0.71). This correlation was not seen in the PS group (r ranging 0.01–0.32) or between growth in the blood and liver in any group (r ranging 0.03–0.38). Bacterial in vivo growth in blood was strongly and negatively correlated with ex vivo_PREBACT_ clearance in the PS group already at 1 h (r = − 0.81), whereas a significant correlation in the ESS group was noted only after 3 h. There was no correlation between ex vivo_PREBACT_ clearance and growth in the organs in any group (data not shown). Ex vivo_POSTBACT_ clearance at 3 h correlated significantly with ex vivo_PREBACT_ at 3 h (ESS group: r = 0.58, *p* = 0.009; PS group: r = 0.62, *p* = 0.014) and with blood bacterial count in both groups, Fig. 3.

## Discussion

In the previous clinical study secondary sepsis was defined as a sepsis starting within 7 days after preceding infection or trauma^9^. Because our animals in the ESS group had their inflammatory response activated only 24 h before the bacterial challenge, we named secondary sepsis in the present study early secondary sepsis. Despite a reduced inflammatory response, the spleen of animals with ESS contained fewer bacteria than animals with PS. A similar trend was seen in the liver and blood in vivo. Thus, the weakened inflammatory response in ESS was not associated with reduced bacterial killing, neither in immune-active organs nor in the blood.

The lower splenic growth in the ESS animals might be caused by reduced splenic uptake from the blood or increased clearance of bacteria. In this model bacteria were negligible in organs other than the spleen and liver (data not shown). In addition, low bacterial counts in the spleen were associated with low counts in the blood, indicating that a reduced uptake is not the mechanism, making increased splenic bacterial killing more plausible. The strong correlation between growth in the spleen and blood might also imply that the increased splenic killing contributes to the lower blood bacterial count seen in the ESS group (Fig. 3). In the spleen bacteria enter an open blood system without an endothelial lining where macrophages reside and surviving bacteria must pass the small endothelial slits of the splenic venous sinusoids before re-entering the circulation^22^. In ESS this trapping mechanism may be more important than other bacterial removal processes. In the liver there is an active uptake of bacteria from the blood^23,24^ and it has recently been shown that this uptake is reduced in secondary sepsis^25^, which might promote a lower bacterial growth in the liver. Particularly noteworthy is that the significant correlation between bacterial growth in the liver and spleen as demonstrated in PS, suggesting related control of the host defence mechanisms, is not observed in ESS despite reduced growth in the spleen and a similar trend in the liver. Dissimilar mechanisms, affected differently by the preceding activated inflammatory response, might constitute an explanation for the lack of correlations between hepatic bacterial growth and that in the spleen and blood in ESS.

Factors other than splenic activity seem to matter to the blood bacterial count in PS. The intrinsic blood bactericidal capacity, as demonstrated by the ex vivo_PREBACT_ bacterial clearance, is the sum of the bactericidal performance of several defence actors, such as activated neutrophils, circulating antimicrobial proteins as well as activation of the complement system, the kallikrein-kinin system and components of the coagulation systems^26–29^. In the PS group high ex vivo_PREBACT_ bacterial clearance appears to be associated with lower levels of bacteraemia, especially during the first hour when this correlation was strong. At 2 and 3 h, this correlation became weaker though still significant. In ESS blood bacterial count was initially more related to splenic killing than blood bactericidal capacity but in contrast to PS, the latter association increased and at 3 h bacterial growth was significantly associated with both the blood intrinsic bactericidal capacity and splenic activity.

While retaining a correlation with the bactericidal capacity before the bacterial challenge, the ex vivo_POSTBACT_ clearance was reduced in both groups suggesting that the components of the antibacterial systems have to some extent been consumed or inhibited after 3 h of bacteraemia. This killing capacity was retained more in the ESS group than in the PS group.

Whereas our results demonstrating increased bacterial killing agree with those in mice pre-treated with endotoxin 24 h or more before the bacterial challenge^6,15,16^, our results with reduced bacterial ex vivo clearance directly after termination of the bacterial infusion seem consistent with the rabbit model^19^. In this rabbit model the bacterial challenge was also given directly after a 1- or 4-h infusion of endotoxin, which could explain the different result of the other studies.

Our findings, together with previous experimental studies on endotoxin tolerance induced 24–72 h before the bacterial challenge^6,15,16^, indicate a decreased inflammatory response associated with increased bacterial killing. In the studies so far, the same results have been obtained both after intravenous and intraperitoneal induction, irrespective of the type of bacterial challenge^6,15^. Because trauma seems to elicit a similar response as endotoxin and infections^30,31^, these results are also likely valid after trauma. Whether this augmented bacterial killing applies later than 72 h, as shown in these studies^15,16^ in the immunosuppressive phase, is not known, warranting further study. However, it might be speculated that there is an evolutionary benefit of protecting animals from additional harm by invading bacteria during the early acute phase of severe trauma or infection. Although extrapolated with caution, the PS model may show similarities to community-acquired sepsis, whereas the ESS model is more comparable with early nosocomial infection in the ICU. Based on our findings, the increased need for a synergistic β-lactam-aminoglycoside combination for the ESS treatment does not seem urgent. Maximum bactericidal antibiotic therapy seems more important in PS. In this connection, a recent retrospective study, restricting patient recruitment to community-acquired bacteraemia demonstrated a better effect by the addition of one single dose of aminoglycoside to the β-lactam treatment than most other comparative studies investigating the effect of this combination^32^.

In contrast to mice, the porcine immune system shares 80% similarity and the physiological responses in sepsis are similar to those in humans^17,18^. The physiognomy is also suitable for intensive care treatment, including sedation, vasopressors and mechanical ventilation that might additionally mitigate the inflammatory response^33–35^, adding strength and clinical relevance to our model. In addition, this model complies with the International Expert Consensus for Pre-Clinical Sepsis Studies^36^.

However, there are some limitations of the model. The challenge of bacteria as an intravenous infusion might clinically resemble the administration of contaminated infusions, which is rare nowadays. The caecal ligation puncture technique has the advantage of resembling the onset of a clinical infection^37^ but is difficult to standardise because of varying numbers and species in the blood and immune-active organs, thus, significantly reducing the power of the study. Using *E. coli* endotoxin followed by an *E. coli* bacterial challenge might be another limitation. However, *E. coli* is the most common Gram-negative bacterial species^38^ and the phenomenon of endotoxin tolerance is not species-specific^8,15^. Only bacterial findings from the spleen and liver were reported in our study. These organs are the major sites that clear bacteria from the bloodstream^22,24,39^, a finding also reported from a porcine model on PS^20^. Cultures were also taken from the kidney and lung in most of our animals. Only occasionally was bacterial growth observed in low numbers, too few to allow a meaningful analysis between the groups.

## Conclusion

Animals with early secondary sepsis exhibit an attenuated inflammatory response as expected but show enhanced antibacterial capacities in the major immune-active organs compared with animals with primary sepsis. During the first hours in early secondary sepsis, blood bacterial count looks to be principally affected by splenic activity and in primary sepsis by the intrinsic blood killing capacity. The results indicate no increased need for synergistic antibiotic combinations in early secondary sepsis.

## Supplementary Information

Supplementary Information.

## Data Availability

The data collected and analysed for the current study are available from the corresponding author on reasonable request.

## References

[1] Singer M (2016). The third international consensus definitions for sepsis and septic shock (sepsis-3). JAMA.

[2] Hotchkiss RS, Monneret G, Payen D (2013). Immunosuppression in sepsis: A novel understanding of the disorder and a new therapeutic approach. Lancet. Infect. Dis.

[3] Otto GP (2011). The late phase of sepsis is characterized by an increased microbiological burden and death rate. Crit. Care.

[4] Torgersen C (2009). Macroscopic postmortem findings in 235 surgical intensive care patients with sepsis. Anesth. Analg..

[5] Castegren M (2012). Differences in organ dysfunction in endotoxin-tolerant pigs under intensive care exposed to a second hit of endotoxin. Shock.

[6] Murphey E, Fang G, Varma TK, Sherwood ER (2007). Improved bacterial clearance and decreased mortality can be induced by LPS tolerance and is not dependent upon IFN-γ. Shock.

[7] Draisma A, Pickkers P, Bouw MP, van der Hoeven JG (2009). Development of endotoxin tolerance in humans in vivo. Crit. Care Med..

[8] Biswas SK, Lopez-Collazo E (2009). Kappa Endotoxin tolerance: New mechanisms, molecules and clinical significance. Trends Immunol..

[9] Castegren M, Jonasson M, Castegren S, Lipcsey M, Sjolin J (2015). Initial levels of organ failure, microbial findings and mortality in intensive care-treated primary, secondary and tertiary sepsis. Crit. Care Resuscit. J. Aust. Acad. Crit. Care Med..

[10] Kumar A (2010). Early combination antibiotic therapy yields improved survival compared with monotherapy in septic shock: A propensity-matched analysis. Crit. Care Med..

[11] Paul M, Lador A, Grozinsky-Glasberg S, Leibovici L (2014). Beta lactam antibiotic monotherapy versus beta lactam-aminoglycoside antibiotic combination therapy for sepsis. Cochr. Database Syst. Rev..

[12] Rhodes A (2017). Surviving sepsis campaign: International guidelines for management of sepsis and septic shock: 2016. Crit. Care Med..

[13] Kalil AC (2018). Infectious Diseases Society of America (IDSA) POSITION STATEMENT: Why IDSA Did Not Endorse the Surviving Sepsis Campaign Guidelines. Clin. Infect. Dis..

[14] Evans L (2021). Surviving sepsis campaign: International guidelines for management of sepsis and septic shock 2021. Intens. Care Med..

[15] Murphey E, Fang G, Sherwood ER (2008). Endotoxin pretreatment improves bacterial clearance and decreases mortality in mice challenged with Staphylococcus aureus. Shock.

[16] Lehner MD (2001). Improved innate immunity of endotoxin-tolerant mice increases resistance to Salmonella enterica serovar typhimurium infection despite attenuated cytokine response. Infect. Immun..

[17] Mair K (2014). The porcine innate immune system: An update. Dev. Comp. Immunol..

[18] Meurens F, Summerfield A, Nauwynck H, Saif L, Gerdts V (2012). The pig: A model for human infectious diseases. Trends Microbiol..

[19] Koch T (1993). Alterations of bacterial clearance induced by endotoxin and tumor necrosis factor. Infect. Immun..

[20] Skorup P (2014). Beneficial antimicrobial effect of the addition of an aminoglycoside to a beta-lactam antibiotic in an *E. coli* porcine intensive care severe sepsis model. PLoS ONE.

[21] Kilkenny C, Browne WJ, Cuthill IC, Emerson M, Altman DG (2010). Improving bioscience research reporting: The ARRIVE guidelines for reporting animal research. PLoS Biol..

[22] Mebius RE, Kraal G (2005). Structure and function of the spleen. Nat. Rev. Immunol..

[23] Parker GA, Picut CA (2005). Liver immunobiology. Toxicol. Pathol..

[24] Yan J, Li S, Li S (2014). The role of the liver in sepsis. Int. Rev. Immunol..

[25] Hanslin K (2019). The impact of the systemic inflammatory response on hepatic bacterial elimination in experimental abdominal sepsis. Intensive Care Med. Exp..

[26] Mayadas TN, Cullere X, Lowell CA (2014). The multifaceted functions of neutrophils. Annu. Rev. Pathol..

[27] Wang G (2014). Human antimicrobial peptides and proteins. Pharmaceuticals.

[28] Dunkelberger JR, Song WC (2010). Complement and its role in innate and adaptive immune responses. Cell Res..

[29] Papareddy P (2010). Proteolysis of human thrombin generates novel host defense peptides. PLoS Pathog..

[30] Menger MD, Vollmar B (2004). Surgical trauma: Hyperinflammation versus immunosuppression?. Langenbecks Arch. Surg..

[31] Stahel PF, Smith WR, Moore EE (2007). Role of biological modifiers regulating the immune response after trauma. Injury.

[32] Liljedahl Prytz K (2020). Antibiotic treatment with one single dose of gentamicin at admittance in addition to a β-lactam antibiotic in the treatment of community-acquired bloodstream infection with sepsis. PLoS ONE.

[33] Sanders RD, Hussell T, Maze M (2009). Sedation & immunomodulation. Crit. Care Clin..

[34] Galley H, DiMatteo M, Webster N (2000). Immunomodulation by anaesthetic, sedative and analgesic agents: Does it matter?. Intensive Care Med..

[35] Wrigge H (2000). Effects of mechanical ventilation on release of cytokines into systemic circulation in patients with normal pulmonary function. Anesthesiology.

[36] Osuchowski MF (2018). Minimum quality threshold in pre-clinical sepsis studies (MQTiPSS): An international expert consensus initiative for improvement of animal modeling in sepsis. Shock.

[37] Piper RD, Cook DJ, Bone RC, Sibbald WJ (1996). Introducing Critical Appraisal to studies of animal models investigating novel therapies in sepsis. Crit. Care Med..

[38] Laupland KB, Church DL (2014). Population-based epidemiology and microbiology of community-onset bloodstream infections. Clin. Microbiol. Rev..

[39] Rogers DE (1960). Host mechanisms which act to remove bacteria from the blood stream. Bacteriol. Rev..

