# Supplementary material for: Enhanced bacterial clearance in early secondary sepsis in a porcine intensive care model
Source: Sci Rep. 2023 Feb 3;13:1964. doi: 10.1038/s41598-023-28880-x (PMC9898276; doi:10.1038/s41598-023-28880-x)
Supplement: Supplementary file 1 — Supplementary Information. [file 41598_2023_28880_MOESM1_ESM.docx]

## Supplemental Digital Contents

**Manuscript Title:** Enhanced Bacterial Clearance in Early Secondary Sepsis in a Porcine Intensive Care Model

## List of Supplemental Digital Contents:

**Supplement 1:** Anaesthesia, ventilation, preparations and intensive care settings

**Supplement 2:** Intensive care treatment protocol

**Supplement 3:** Organism preparation

**Supplement 4:** Bacterial culturing of blood samples, organ biopsies and *ex vivo* investigations

**Supplement 5:** Cytokine analyses and organ function evaluation

## Supplemental Digital Content 1:

### Anaesthesia, ventilation, preparations and intensive care settings

The animals had unlimited access to food and water until 1 h before the experiment and arrived at the research unit in the morning. All preparations were done under aseptic conditions. General anaesthesia was induced by an intramuscular injection of 6 mg x kg^-1^ tiletamine/zolazepam combined with 2.2 mg x kg^-1^ xylazine. Venous access was established in auricular veins and an intravenous (iv) injection of 20 mg morphine and 100 mg ketamine was injected before tracheotomy. An initial bolus of acetated Ringer’s solution 20 mL x kg^-1^ was given. Catheterisation of a superior caval vein, a pulmonary artery (Swan Ganz catheter) and a cervical artery were performed. An indwelling urinary catheter was inserted into the bladder through vesicostomy. After preparations the animals were placed on their sides and underwent at least 30 min of stabilisation after completed preparations. Temperature was measured using the thermistor in the Swan-Ganz catheter. To compensate heat losses a heating pad set to 38°C, was situated under the animals for the first 6 h if their body core temperature remained <42°C.

During the experiment, the animals were continuously sedated iv with 8 mg x kg^-1^ x h^-1^ sodium pentobarbital and 0.48 mg x kg^-1^ x h^-1^ morphine dissolved in a 2.5% glucose solution at 8 mL x kg-1 x h-1 and muscle-relaxed with rocuronium bromide 2.5 mg x kg^-1^ x h^-1^. Continuous fluid replacement of acetated Ringer’s solution was administered iv at 2 mL x kg^-1^ x h^-1^, resulting in a total fluid administration rate of 10 mL x kg^-1^ x h^-1^during the entire experiment.

The animals were mechanically ventilated using a Servo I^®^ ventilator. Respiratory settings were as follows: volume-controlled mode, inspired oxygen fraction in air (FiO_2_) 30%, respiratory rate 25 min^-1^ and positive end-expiratory pressure (PEEP) 5 cm H_2_O. Tidal volume was adjusted during the upstart period to maintain an arterial carbon dioxide pressure (PaCO_2_) of 5.0-5.5 kPa. To prevent and treat eventual atelectasis the animals’ body position were changed and alveolar recruitment manoeuvres were performed every 6 h in the ESS animals. Every effort was made to minimize suffering and at animal signs of not being sedated deep enough during anaesthesia, an iv bolus of 100 mg ketamine was injected, and if a reaction to pain stimuli of the fore hoof was observed, 10 mg morphine iv was provided.

## Supplemental Digital Content 2

### Intensive care treatment protocol

The intensive care setting was maintained with interventions following a treatment protocol to keep the vital signs within pre-set limits according to the Table below.

| **Parameter** | **Threshold values for intervention** | **Intervention** |
| --- | --- | --- |
| PaO_2_ | <10 kPa first time | Increase FiO_2_ to 0.6 |
|  | <10 kPa thereafter | 1. Increase FiO_2_ to the next level 0.6 🡪0.8 🡪1.0  AND  2. Increase PEEP to the next level 5🡪8🡪10🡪14 cmH_2_0  AND  3. Alveolar recruitment^a^ |
|  | >20 kPa | Decrease FiO_2_ to the next level 1.0 🡪0.8 🡪 0.6 🡪0.3 |
| PaCO_2_ | >6.5 kPa | Increase tidal volume by 10% up to 15 ml x kg^-1^ |
|  | <4.5 kPa | If RR ≤25, decrease tidal volume by 10% down to 4 ml x kg^-1^  If RR >25 Decrease RR by 10% |
| P pause | >30 cmH_2_O | Decrease tidal volume to 7 ml/kg and increase RR to the same minute volume. |
|  | >30 cmH_2_O persistently | I:E is set to 1:1 |
| MAP | MAP=MPAP at < 90 min after start of endotoxin or bacterial infusion. | Single dose of 40 µg of norepinephrine |
|  | MAP=MPAP at > 90 min | 1. Single dose of 20 µg of norepinephrine  AND 2. Start norepinephrine infusion 0.07 µg x kg^-1^x min^-1^. If ongoing, in-crease rate one step: 0.07🡪 0.13🡪 0.29🡪 0.54 µg x kg^-1^ x min^-1^  AND  3. Single bolus dose of RA 15 ml x kg^-1^ |
|  | <60 mmHg (50 is used as a threshold at < 90 min after start of endotoxin or bacterial infusion) | Start norepinephrine infusion 0.07 µg x kg^-1^ x min^-1^. If ongoing, increase rate one step: 0.07🡪 0.13🡪 0.29🡪 0.54 µg x kg^-1^ x min^-1^  If CO <2.5 L/min give RA bolus 15 ml x kg^-1^ |
|  | >100 mmHg | Decrease norepinephrine to the next step 0.54🡪 0.29🡪 0.13🡪 0.07🡪0 µg x kg^-1^ x min^-1^ |
| CO | <2 L/min | 1. Start norepinephrine infusion 0.07 µg x kg^-1^ x min^-1^. If ongoing, increase rate one step: 0.07🡪 0.13🡪 0.29🡪 0.54 µg x kg^-1^ x min^-1^  AND/OR  2. Single bolus dose of RA 15 ml x kg^-1^ |
| B-glucose | <4.0 mmol/L | Single dose of 20 ml 30% glucose iv |

CO= cardiac output, FiO_2_= inspired fraction of oxygen, I:E= Inspiratory:Expiratory ratio, MAP= mean arterial pressure, min= minutes, MPAP= mean pulmonary arterial pressure

PaO_2_= arterial partial pressure of oxygen, PEEP= positive end expiratory pressure, RA= Ringer acetate, RR= respiratory rate

^a^ PEEP was increased stepwise until a peak pressure of 35 cm H_2_O was reached. At this point, an inspiratory hold was performed for 10 seconds. Thereafter, the PEEP was stepwise decreased to the PEEP defined by the protocol. If MAP decreased to the level of the MPAP, the recruitment maneuverer was aborted.

## Supplemental Digital Content 3

### Organism preparation

Before the experiment, the *E. coli* were harvested, reinoculated on a cysteine lactose electrolyte deficient plate (CLED) and cultured overnight in 37^o^ C in an incubation chamber. Two hours before each experiment the bacteria were grown to logarithmic growth phase in lysogeny broth medium according to Miller (LB). Thereafter, a 2 min centrifugation at 6000 rpm at 22°C enabled LB removal and resuspension in phosphate-buffered saline and then the concentration was assessed using spectrophotometry. At baseline, all groups received an infusion of 8.3 log_10_ colony forming units (CFU) x h^-1^ for 3 h.

## Supplemental Digital Content 4

### Bacterial culturing of blood samples, organ biopsies and *ex vivo* investigations

#### In vivo blood and organ bacterial cultures

Bacterial count in arterial blood was investigated before baseline and then hourly by cultivation of 0.1 ml in triplicate on CLED plates in all animals. In the ESS group blood samples at -24 h were also obtained to ensure that no bacteremia was present at the start. Quantification was determined by the viable count technique with a detection limit in blood of 5 CFU x mL^-1^.

Post-mortem organ biopsies of approximately 1-2 g from the liver and spleen were taken under aseptic conditions and placed in 3 mL phosphate-buffered saline and homogenised for 4 min in either a Stomacher^®^ 80 Biomaster or in glass vessel tissue grinders of Potter-Elvehjem type. From these solutions 0.2 ml were plated in triplicate on CLED plates for quantification by viable count. The detection limit in organs was 5 CFU x g^-1^.

*Ex vivo bactericidal capacity of blood*

Arterial lithium-heparinised blood was obtained to measure bacterial killing capacity of blood ex vivo at baseline before bacteria infusion (*Ex vivo*_PREBACT_) and 15 min after termination of the bacterial infusion (*Ex vivo*_POSTBACT_). The blood was inoculated with 10^5^ CFU x mL^-1^ *E. coli* in duplicate and incubated at 37°C. Samples of 0.2 mL was collected at 3 h and 6 h from the *Ex vivo*_PREBACT_ and at 3 h in the *Ex vivo*_POSTBACT_ for viable count performed as serial dilutions and inoculation to CLED plates.

## Supplemental Digital Content 5

**Cytokine analyses and organ function evaluation**

*Cytokines analyses*

To assess the inflammatory response tumour necrosis factor alpha (TNF-α) and interleukin-6 (IL-6) were analysed in arterial plasma at baseline and then hourly. TNF-α and IL-6 were determined by commercial porcine-specific sandwich enzyme-linked immunosorbent assays (DY690B, (TNF-α) and DY686, (IL-6), R&D Systems, Minneapolis, MN, USA).

*Organ function evaluation*

In the ESS group physiological data and blood gases were registered at -24 h and thereafter at -22 h, at -18 h and on every third hour until baseline. At baseline and hourly after that, both groups underwent blood sampling and registration of physiological data to optimise the intensive care treatment (Supplemental Digital Content 2) and evaluate organ function using the Sequential Organ Failure Assessment (SOFA) score [1]**.** The SOFA score was employed to evaluate if a sepsis reaction occurred as assessed by standard parameters: respiration (P/F ratio), coagulation (platelets), liver (bilirubin), cardiovascular (MAP and need for vasoactive drugs) and renal (creatinine and urine output), but without assessment of the central nervous system because of the sedation.

Respiratory pressures and set values from ventilator readings as well as measurements of MPAP were monitored continuously. Cardiac output was assessed by the thermodilution method using the thermistor in the Swan-Ganz catheter. The amount of delivered norepinephrine and urinary output were registered hourly. At baseline and every hour during the experiment, arterial cervical blood and central venous blood samples were analysed for gas tensions (PaO_2_, PaCO_2_) and glucose on an ABL^®^ 800 and a Hemoximeter ^®^ OSM-3 (Radiometer, Brønhøj, Denmark). Blood or plasma samples were also analysed for blood cell count on a Cell Dyn Sapphire cell counter (Abbott Scandinavia) and for bilirubin (reagent 6L45) and creatinine (reagent 8L24) on an Architect ^®^ Ci8200 analyser (Abbott Scandinavia).

## References

1. Singer, M. *et al.* The third international consensus definitions for sepsis and septic shock (sepsis-3). *JAMA* **315**, 801-810 (2016).
